# Indomethacin treatment prior to pentylenetetrazole-induced seizures downregulates the expression of *il1b* and *cox2* and decreases seizure-like behavior in zebrafish larvae

**DOI:** 10.1186/s12868-016-0246-y

**Published:** 2016-03-09

**Authors:** Patrícia Gonçalves Barbalho, Iscia Lopes-Cendes, Claudia Vianna Maurer-Morelli

**Affiliations:** Laboratory of Zebrafish, Department of Medical Genetics - School of Medical Sciences, University of Campinas (UNICAMP), Tessália Vieira de Camargo, 126 Cidade Universitaria “Zeferino Vaz”, Campinas, SP 13083-887 Brazil; Laboratory of Molecular Genetics, Department of Medical Genetics - School of Medical Sciences, University of Campinas (UNICAMP), Tessália Vieira de Camargo, 126 Cidade Universitaria “Zeferino Vaz”, Campinas, SP 13083-887 Brazil

**Keywords:** Seizure, Zebrafish, Pentylenetetrazol, Interleukin-1 beta, Cyclooxygenase-2, Indomethacin, Inflammation

## Abstract

**Background:**

It has been demonstrated that the zebrafish model of pentylenetetrazole (PTZ)-evoked seizures and the well-established rodent models of epilepsy are similar pertaining to behavior, electrographic features, and *c*-*fos* expression. Although this zebrafish model is suitable for studying seizures, to date, inflammatory response after seizures has not been investigated using this model. Because a relationship between epilepsy and inflammation has been established, in the present study we investigated the transcript levels of the proinflammatory cytokines interleukin-1 beta (*il1b*) and cyclooxygenase-2 (*cox2a* and *cox2b*) after PTZ-induced seizures in the brain of zebrafish 7 days post fertilization. Furthermore, we exposed the fish to the nonsteroidal anti-inflammatory drug indomethacin prior to PTZ, and we measured its effect on seizure latency, number of seizure behaviors, and mRNA expression of *il1b*, *cox2b,* and *c*-*fos*. We used quantitative real-time PCR to assess the mRNA expression of *il1b, cox2a, cox2b,* and *c*-*fos*, and visual inspection was used to monitor seizure latency and the number of seizure-like behaviors.

**Results:**

We found a short-term upregulation of *il1b*, and we revealed that *cox2b*, but not *cox2a*, was induced after seizures. Indomethacin treatment prior to PTZ-induced seizures downregulated the mRNA expression of *il1b*, *cox2b,* and *c*-*fos*. Moreover, we observed that in larvae exposed to indomethacin, seizure latency increased and the number of seizure-like behaviors decreased.

**Conclusions:**

This is the first study showing that *il1b* and *cox*-*2* transcripts are upregulated following PTZ-induced seizures in zebrafish. In addition, we demonstrated the anticonvulsant effect of indomethacin based on (1) the inhibition of PTZ-induced *c*-*fos* transcription, (2) increase in seizure latency, and (3) decrease in the number of seizure-like behaviors. Furthermore, anti-inflammatory effect of indomethacin is clearly demonstrated by the downregulation of the mRNA expression of *il1b* and *cox2b*. Our results are supported by previous evidences suggesting that zebrafish is a suitable alternative for studying inflammation, seizures, and the effect of anti-inflammatory compounds on seizure suppression.

## Background

Zebrafish experimental models are now widely accepted for investigating human diseases, including epilepsy [1–4]. Importance of this animal model is mainly based on its remarkable features combining exceptionally simple genetic manipulations, which are ideal for forward and reverse genetic investigations, and easy phenotype assessment in a short period of time. Other advantages of this fish species are as follows: low maintenance cost, easy breeding, high fecundity, external fertilization and development, short generation time, and transparency during embryonic and larval stage. Furthermore, their genome shares approximately 70 % homology with the human genome comprising large regions of conserved synteny [5].

Zebrafish exposed to chemoconvulsant drugs mimic behavior, electrographic findings, and upregulation of *c*-*fos* in brain regions related to neuronal activation [3, 4, 6]. Zebrafish are sensitive to common anticonvulsant drugs; therefore, they are widely used for the high throughput screening of novel antiepileptic drugs (AEDs) [7–13].

Evidence obtained using hippocampal surgical specimens from patients with pharmacoresistant epilepsy and experimental rodent models demonstrated that proinflammatory cytokines and inflammatory mediators are upregulated after seizures, suggesting that the inflammatory response may play an important role in the pathophysiology of epilepsy [14–18]. Among the proinflammatory cytokines, interleukin-1 beta (IL-1β) is the most widely investigated. IL-1β exerts its action by binding to the IL-1 receptor, which initiates a downstream signaling process that activates the transcription factor nuclear factor-κB (NF-κB). Activation of NF-κB leads to the transcription of multiple inflammation-associated genes, including cyclooxygenase (COX)-2 [19–22]. COX-2 is a key enzyme responsible for the conversion of arachidonic acid into prostaglandins, potent mediators of inflammatory signaling [19–22]. One of these prostaglandins converted by COX-2 is prostaglandin E2 (PGE2), which upregulates the expression of IL-1β [19–24].

Because a relationship between epilepsy and inflammation has been established and inflammatory response in the pentylenetetrazole (PTZ)-seizure model has not been studied, we investigated the expression of the *il1b* and *cox2* transcripts in zebrafish after seizures. In addition, we measured the effects of indomethacin, a nonsteroidal antiinflammatory drug, on seizure latency, number of seizure-like behaviors, and *c*-*fos* expression used as a marker of neuronal activity [10].

## Results

### Temporal expression of *il1b* in the brain of zebrafish larvae after PTZ-induced seizures

**Fig. 1:**
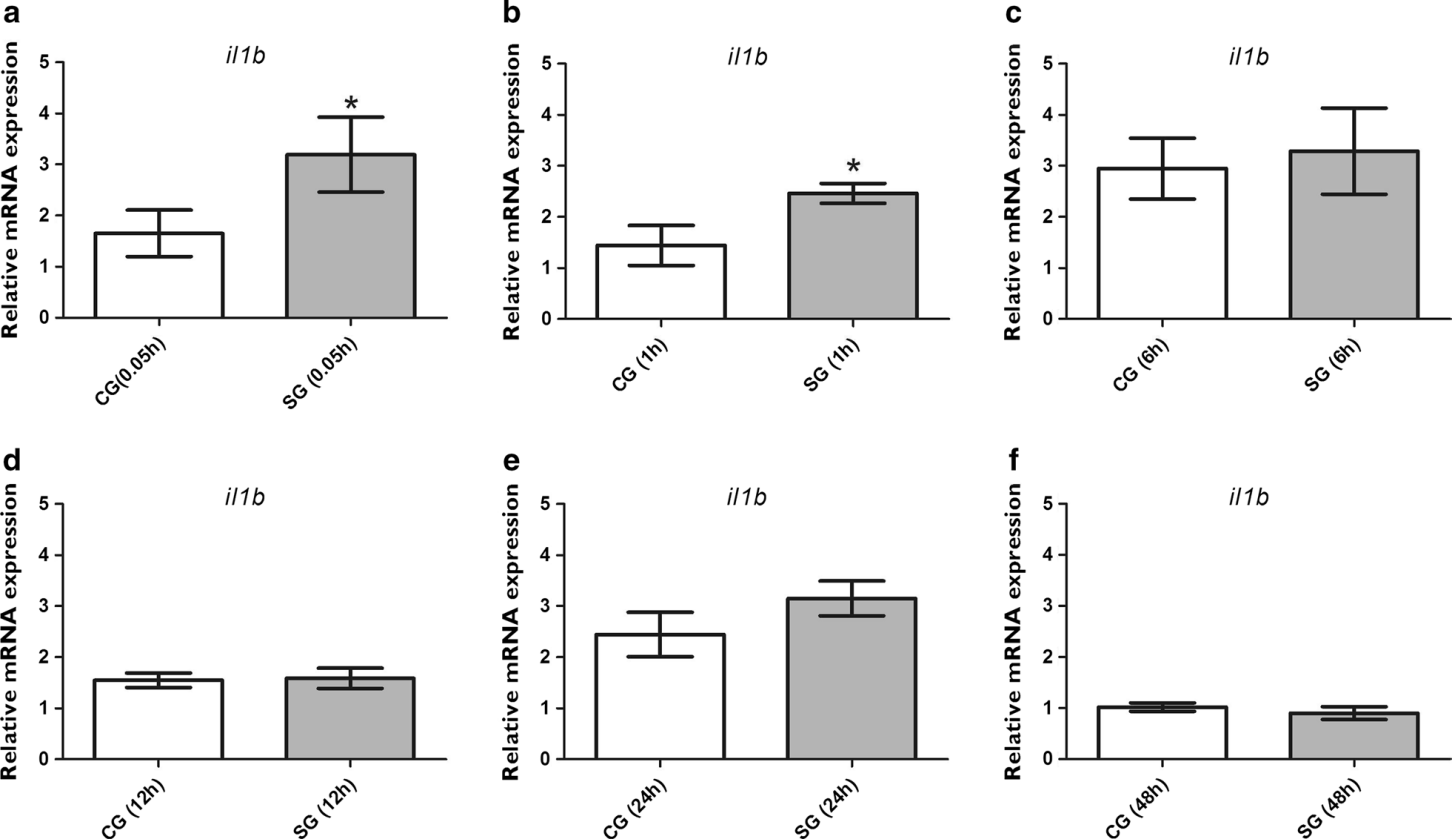
Temporal expression profile of *il1b* in the brain of zebrafish after pentylenetetrazole-evoked seizures. Relative quantification of the interleukin-1 beta (*il1b)* transcript 0.05, 1, 6, 12, 24, and 48 h after pentylenetetrazole (PTZ)-induced seizures in the brain of zebrafish at 7 days post fertilization. Each time-point seizure group was exposed to 15 mM PTZ for 20 min, and their time-matched control groups were handled identically, but included exposure to water (*n* = 5 per group). Data obtained from each seizure group was compared with their respective time-point matched control group. Data are presented as mean ± SEM. Statistical analyses were performed using the Mann–Whitney test, and differences were considered significant if p < 0.05. *Asterisk* (*) indicates p ≤ 0.05. *CG* control group, *SG* seizure group

Temporal expression profile of *il1b* was analyzed 0.05, 1, 6, 12, 24, and 48 h after PTZ-induced seizures by comparing the control (CG) and seizure (SG) groups at each time point. We found a short-term upregulation of *i11b* mRNA levels 0.05 h (p = 0.02) and 1 h (p = 0.02) after seizures (Fig. 1). However, no significant differences were found between the CG and SG 6, 12, 24, or 48 h after seizures (p > 0.05) (Fig. 1).

### The mRNA expression of *cox2a* and *cox2b* in the brain of zebrafish larvae after PTZ-induced seizures

**Fig. 2:**
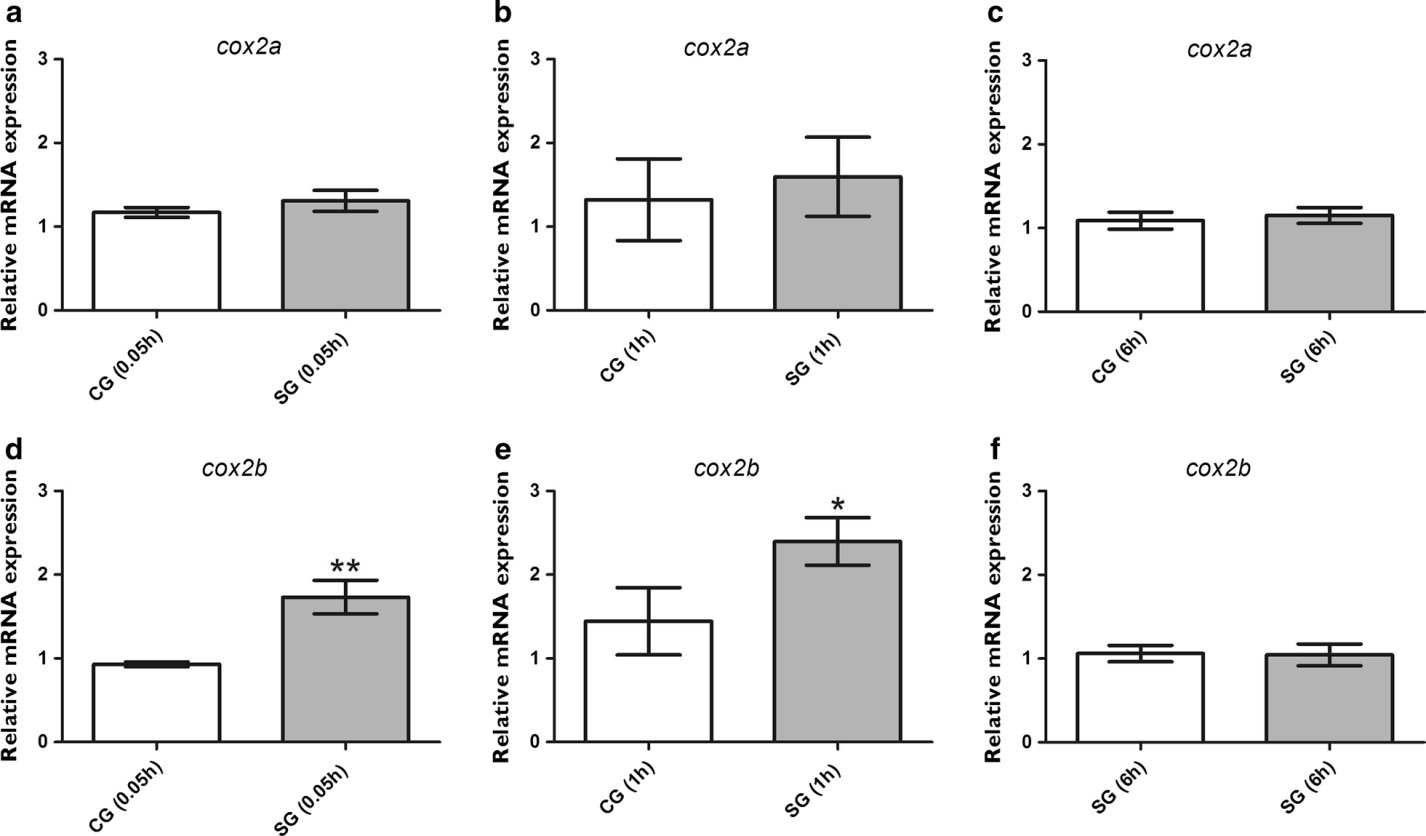
*cox2a* and *cox2b* expression in the brain of zebrafish after pentylenetetrazole-evoked seizures. Relative quantification of cyclooxygenase 2 a and b (*cox2a* and *cox2b)* transcripts 0.05, 1, and 6 h after pentylenetetrazole (PTZ)-induced seizures in the brain of zebrafish at 7 days post fertilization. Each time-point seizure group was exposed to 15 mM PTZ for 20 min, and their time-matched control groups were handled identically, but included exposure to water (*n* = 5 per group). Data obtained from each seizure group was compared with their respective time-point matched control group. Data are presented as mean ± SEM. Statistical analyses were performed using the Mann–Whitney, and differences were considered significant if p < 0.05. *One asterisk* (*) indicated that p ≤ 0.05; *two asterisks* (**) indicated that p ≤ 0.01. *CG* control group, *SG* seizure group

Because no significant differences were found in the mRNA expression of *il1b* pertaining to longer time periods, we chose to evaluate the temporal expression profile of *cox2a* and *cox2b* 0.05, 1, and 6 h after PTZ-induced seizures. Our results showed that both *cox2a* and *cox2b* were constitutively expressed in the CG (Fig. 2a–e); however, after PTZ exposure, the expression pattern of these genes showed differences. Animals in the CG and SG had similar *cox2a* mRNA levels after seizures (p > 0.05; Fig. 2a–c). However, c*ox2b* mRNA levels were upregulated 0.05 h (p = 0.004) and 1 h (p = 0.008) after seizures in the SG compared with the corresponding data in the CGs (Fig. 2d, e). No statistical significance was found 6 h after seizures (p = 0.27; Fig. 2f).

### Effect of indomethacin administered prior to PTZ on the mRNA expression levels of *il1b,cox2b,* and *c*-*fos*

**Fig. 3:**
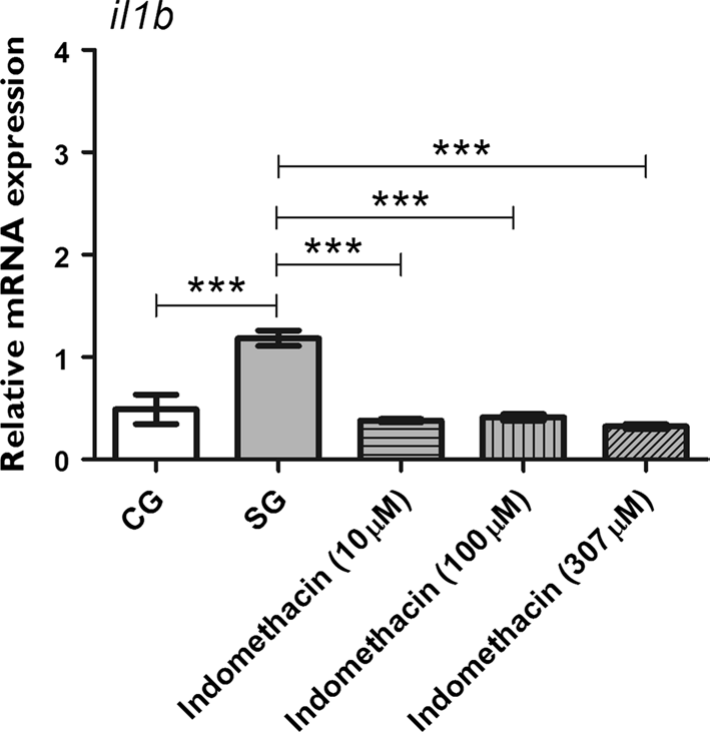
Indomethacin, administered prior to pentylenetetrazole-induced seizures, on *il1b* level in the brain of zebrafish. Relative quantification of interleukin-1 beta (*il1b)* mRNA expression level 0.05 h after pentylenetetrazole (PTZ)-induced seizures in the brain of zebrafish at 7 days post fertilization. Seizure group (SG) comprised animals exposed to 15 mM PTZ for 20 min. Indomethacin groups (10, 100, and 307 μM) comprised animals that received indomethacin treatment prior to PTZ. Animals in the control group (CG) were handled identically, but included exposure to water (no PTZ or indomethacin treatments; *n* = 5 per group). Data are presented as mean ± SEM. Statistical analysis was performed by one-way analysis of variance (ANOVA) with Bonferroni’s post hoc test. *Three asterisks* (***) indicate that p ≤ 0.001. *CG* control group, *SG* seizure group

**Fig. 4:**
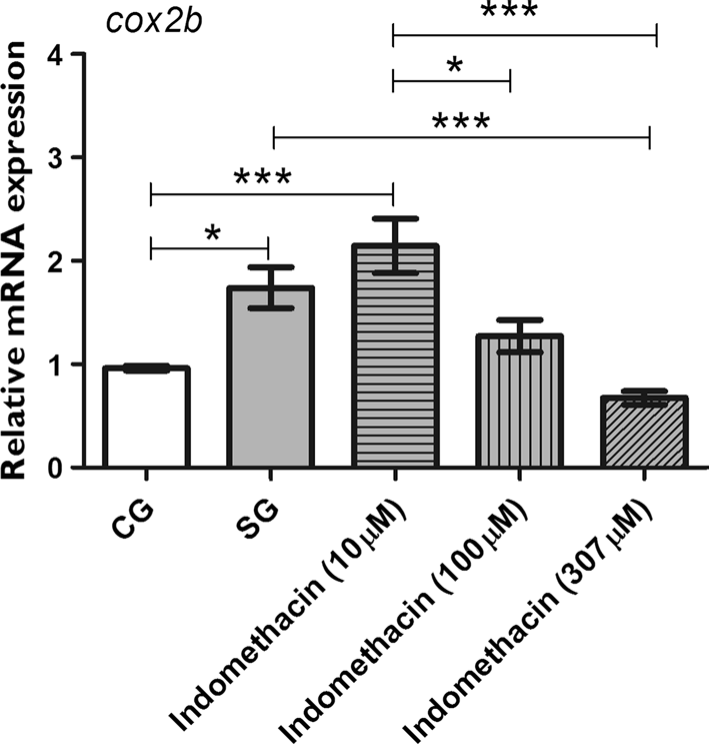
Indomethacin, administered prior to pentylenetetrazole-induced seizures, on *cox2b* level in the brain of zebrafish. Relative quantification of cyclooxygenase 2 b (*cox2b*) mRNA expression level 0.05 h after pentylenetetrazole (PTZ)-induced seizures in the brain of zebrafish at 7 days post fertilization. Seizure group (SG) was composed of animals exposed to 15 mM PTZ for 20 min. Indomethacin groups (10, 100, and 307 μM) were composed of animals that received indomethacin treatment prior to PTZ. Animals in the control group (CG) were handled identically, but they were treated with water (no PTZ and no indomethacin treatments; *n* = 5 per group). Data are presented as mean ± SEM. Statistical analysis was performed by one-way analysis of variance (ANOVA) with Bonferroni’s post hoc test. *One asterisk* (*) indicated that p ≤ 0.05; *two asterisks* (**) indicated that p ≤ 0.01; *three asterisks* (***) indicated that that p ≤ 0.001. *CG* control group, *SG* seizure group

**Fig. 5:**
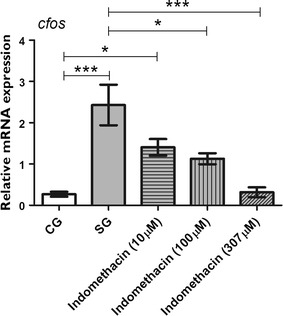
Effect of indomethacin exposure, prior to pentylenetetrazole-induced seizures, on *c*-*fos* level of the zebrafish brain. Relative quantification of *c*-*fos* transcript levels 0.05 h after pentylenetetrazole (PTZ)-induced seizures in the brain of zebrafish at 7 days post-fertilization. Seizure group was composed of animals exposed to 15 mM PTZ for 20 min. Indomethacin groups (10, 100, and 307 μM) were composed of animals that received indomethacin treatment prior to PTZ. Animals in the control group were handled identically, but included exposure to water (no PTZ or indomethacin treatments; *n* = 5 per group). Data are presented as mean ± SEM. Statistical analysis was performed by one-way analysis of variance (ANOVA) with Bonferroni’s post hoc test. *One asterisk* (*) indicated that p ≤ 0.05; *two asterisks* (**) indicated that p ≤ 0.01; *three asterisks* (***) indicated that that p ≤ 0.001. *CG* control group, *SG* seizure group

We used indomethacin at three different concentrations (10, 100, or 307 μM) prior to seizure-induction, and we quantified the mRNA levels of *il1b*, *cox2b,* and *cfos* 0.05 h after PTZ-induced seizures, when the transcript levels of *il1b* and *cox2b* are the highest. Our results revealed that indomethacin treatment prior to PTZ-induced seizures downregulated the mRNA expression of *il1b*, *cox2b,* and *c*-*fos.* As shown in Fig. 3, expression of *il1b* was downregulated by indomethacin when we compared the SG with all indomethacin concentration tested (p ≤ 0.001). In addition, *il1b* mRNA levels in the indomethacin-treated groups were similar to that in the CG. No significant difference was found between indomethacin treatment groups (Fig. 3). The mRNA expression of *cox2b* was upregulated in the SG (p ≤ 0.05) and 10 μM indomethacin group (p ≤ 0.001) when both were compared to the CG (Fig. 4). Indomethacin treatment at 307 μM significantly downregulated the mRNA expression of *cox2b* compared with the SG (p ≤ 0.001; Fig. 4). Comparisons between indomethacin treatments groups showed that c*ox2b* mRNA levels were downregulated at 100 μM (p ≤ 0.05) and 307 μM (p ≤ 0.001) when compared to 10 μM (Fig. 4). Transcript level of *c*-*fos* was upregulated in the SG (p ≤ 0.001) and 10 μM indomethacin pretreated group (p ≤ 0.05) when compared to the CG (Fig. 5). However, 100 μM (p ≤ 0.05) and 307 μM (p ≤ 0.001) indomethacin treatment downregulated the c*fos* mRNA level when compared to the SG (Fig. 5).

### Seizure onset latency and the number of seizure-like behaviors

**Fig. 6:**
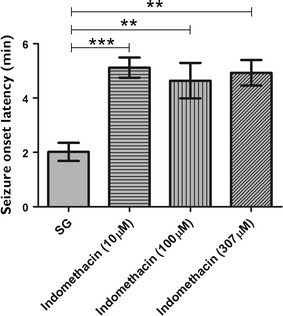
Effect of indomethacin treatment prior to pentylenetetrazole induced seizures on seizure latency. Animals were exposed to 10, 100, or 307 μM indomethacin for 24 h prior to pentylenetetrazole (PTZ)-induced seizures, and latency of the first seizure-like behavior (stage 3, equivalent to loss of body posture) was evaluated following visual observation. The seizure group (SG) was composed of animals that were exposed to 15 mM PTZ (n = 6 per group). Data are presented as mean ± SEM. Statistical analysis was performed by one-way analysis of variance (ANOVA) with Bonferroni’s post hoc test. *One asterisk* (*) indicated that p ≤ 0.05; *two asterisks* (**) indicated that p ≤ 0.01; *three asterisks* (***) indicated that that p ≤ 0.001

**Fig. 7:**
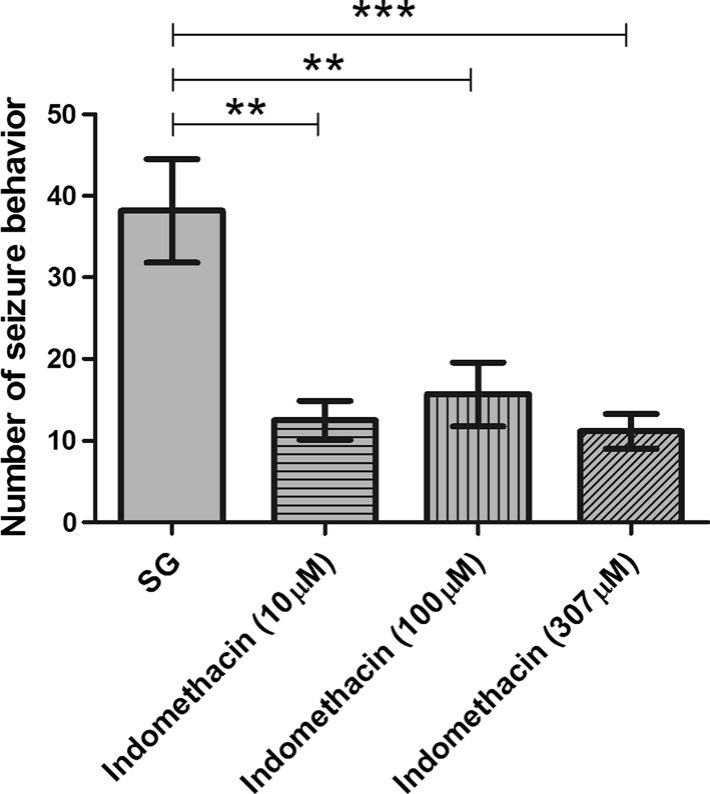
Effect of indomethacin treatment prior to pentylenetetrazole-induced seizures on the number of seizure-like behaviors. Animals were exposed to 10, 100, or 307 μM indomethacin prior to pentylenetetrazole (PTZ)-induced seizures. Number of seizure-like behaviors was evaluated following visual observation during the 20 min of PTZ (15 mM) exposure. Seizure-like behavior was registered if the zebrafish larvae lost their body posture (stage 3). Data are presented as mean ± SEM (n = 6 per group). Statistical analysis was performed by one-way analysis of variance (ANOVA) with Bonferroni’s post hoc test. *One asterisk* (*) indicated that p ≤ 0.05; *two asterisks* (**) indicated that p ≤ 0.01; *three asterisks* (***) indicated that that p ≤ 0.001. *SG* seizure group

We evaluated the effect of indomethacin administered prior to PTZ-induced seizures by analyzing seizure onset latency (stage 3, equivalent to the loss of body posture) and the number of seizure-like behaviors. Each larva was observed individually under each experimental condition. In animals pretreated with 10, 100, or 307 μM indomethacin, we found a significant increase of onset latency (give in minutes) compared with animals in the SG (p ≤ 0.001, p ≤ 0.01, and p ≤ 0.01, respectively; Fig. 6). Furthermore, all indomethacin concentrations tested reduced the number of seizure-like behaviors compared with the PTZ treatment alone (10 μM, p ≤ 0.01; 100 μM, p ≤ 0.01; and 307 μM, p ≤ 0.001; Fig. 7).

## Discussion

It has been demonstrated previously that seizures induce the upregulation of IL-1β and COX-2 in clinical specimens and experimental models of epilepsy [14–18]; however, to reveal the main role of inflammatory molecules in epilepsy, further investigations are necessary. Rodent models of epilepsy are widely used in experimental research, but due to its several favorable characteristics, zebrafish seizure models can significantly contribute to understanding the role of inflammation following seizures. In addition, zebrafish models of human diseases are particularly suitable for the pharmacological testing of drugs in a convenient way. Immune and inflammatory responses in zebrafish are comparable to those found in mammals [25–28]; however, to our knowledge, no data is available about the expression of inflammatory biomarkers such as *il1b* and *cox2* in zebrafish seizure models.

Our results clearly showed that the expression of *il1b* is upregulated shortly after seizures in the larval brain (Fig. 1). A previous study of Minami et al. [29] showed that the mRNA levels of IL-1β increase more rapidly when the seizure was induced by PTZ compared with kainic acid in rodents. Recently, Järvelä et al. [30] reported that the mRNA level of IL-1β is elevated for up to 24 h after *status epilepticus* (SE) induced by kainic acid in rodents. Our finding suggests that the transcription profile of *il1b* after PTZ exposure in our zebrafish model is similar to that found in a rodent model of PTZ-induced seizures.

Because of evolutionary gene duplication, the zebrafish genome contains two functional *cox2* genes termed as c*ox2a* and *cox2b* [31, 32]. The mRNAs of *cox2a* and *cox2b* are constitutively expressed in numerous tissues, including the zebrafish brain [31]. In this study, we investigated the inducible expression of both *cox2* genes after PTZ-induced seizures. Our results revealed a characteristic transcriptional response in both genes. Expression of *cox2b*, but not *cox2a*, is upregulated immediately after PTZ-induced seizures (0.05 h) and 1 h after the seizure in the brain of zebrafish (Fig. 2a, b). It is important to note that a study by Ishikawa et al. [31] showed that *cox2b* is more similar in structure to the mammalian *cox2* than *cox2a*, which may explain our results [31].

Because the mRNA levels of *il1b* and *cox2b* were upregulated after seizures, we investigated the effect of an anti-inflammatory drug administered prior to PTZ-induced seizures. Our results showed that indomethacin used at various concentrations downregulated the expression of *il1b* (Fig. 3). This result is similar to the data obtained using the pilocarpine-induced model of epilepsy, wherein indomethacin administration prior to pilocarpine injection downregulates the expression of IL-1β in rats [33]. In addition, we showed that indomethacin significantly downregulated the mRNA expression of *cox2b*, but only at a concentration of 307 μM.

After showing that indomethacin was able to reduce the mRNA levels of *il1b* and *cox2b*, we investigated if indomethacin reduces the convulsant effect of PTZ (Fig. 4). Therefore, we assessed neuronal activity by measuring the mRNA expression of *c*-*fos*, and we investigated seizure behavior during PTZ exposure by analyzing the latency of seizure onset and the number of seizure-like behaviors, which are both well-known characteristics of seizures according to the literature [3]. The *c*-*fos* gene is a proto–oncogene, which is expressed rapidly and transiently in neurons following neuronal excitation such as that in seizures; therefore, expression of *c*-*fos* has been widely used as a marker for neuronal activity [10]. Baxendale et al. [10] showed that *c*-*fos* is a sensitive marker for investigating anticonvulsant properties of several pharmacological compounds. Our results showed that indomethacin downregulated *c*-*fos* expression at 100 and 307 μM (Fig. 5).

Furthermore, indomethacin administered prior to PTZ reduced the behavioral signs of seizure because it both increased seizure latency (time to reach stage 3, which is characterized by the loss of body posture) (Fig. 6) and decreased the number of seizure-like behaviors for all indomethacin concentrations used in this study compared with the untreated group (Fig. 7).

Although our results clearly suggest that indomethacin has significant effects on several parameters related to seizure activity, we did not demonstrate that the anticonvulsant effect of indomethacin is caused exclusively by its anti-inflammatory action on *il1b* and *cox2b*; therefore, further studies are necessary to address this question.

## Conclusions

We have shown for the first time that transcriptional levels of two important inflammatory biomarkers related to epilepsy, *il1b* and *cox2,* are upregulated in the brain of zebrafish after PTZ-induced seizures. Furthermore, we found that indomethacin exposure prior to PTZ-induced seizures had an anti-inflammatory effect by reducing the mRNA expression of *il1b,cox2b,* and *c*-*fos*, and by increasing seizure latency and decreasing the number of seizure-like behaviors.

Taken together, our results demonstrate that the zebrafish seizure model is a valuable alternative model for studying the molecular mechanisms of inflammation and seizures and for the investigation of anti-inflammatory compounds that may have a potential therapeutic effect in seizure suppression.

## Methods

### Animals

Wild-type zebrafish (adults, larvae, and embryos) were maintained according to standard procedures [34]. Adult fish were housed in 30–50 L tanks (two animals per liter) filled with non-chlorinated water cleared with mechanical and chemical filtration. Adult fish were maintained at 26 ± 2 °C and in a simulated photoperiod cycle of 10 h dark/14 h light. Adult fish were fed twice a day with commercial flake fish food (Tetramin, Tetra, Blacksburg, VA) and once a day with artemia; larvae were fed with paramecium and artemia twice a day. Fertilized eggs were collected after natural spawning. Embryos and larvae were housed using Petri dishes filled with water in an incubator system at the same temperature and photoperiods that were used for maintaining the adults. Larvae were staged according to the morphological criteria established by Kimmel et al. [35]. All zebrafish experiments were approved by the Ethical Committee for Animal Research of the University of Campinas (protocol number 3098-1).

### Pentylenetetrazole treatment

Larvae at 7 days post fertilization (dpf) were separated in the seizure (SG) and control (CG) groups. Larvae in the SG were placed in a 24-well plate (one larvae per well) containing 15 mM PTZ (Sigma-Aldrich, St. Louis, MO, USA), a γ-aminobutyric acid (GABA)_A_ antagonist, for 20 min [3]. After PTZ exposure, animals were washed to eliminate the residual PTZ before being transferred into petri dishes. Fish in the CG were handled identically; however, water was used instead of PTZ. It is important to highlight that each CG or SG presented in this study was composed by a different set of animals.

### Monitoring of seizure-like behavior

Seizure-like behavior of the larvae was monitored by visual inspection and was classified based on the criteria established in a previous study [3]. To visualize the swimming behavior of larvae, we used the Stereomaster^®^ microscope (Fisher Scientific, Waltham, MA). Briefly, classification was performed according to the following criteria: stage 1, increased swimming activity; stage 2, rapid circular “whirlpool-like” swimming; and stage 3, seizure-like activity progressing to clonus-like convulsions followed by a brief loss of posture. Animals included in this study exhibited these well-defined behavioral patterns during PTZ exposure. Moreover, we evaluated the latency of seizure onset and the number of seizures during PTZ exposure. It is important to emphasize that latency was determined as the period between the start of PTZ exposure and the appearance of stage 3 seizure-like behavior, and the number of seizure-like behaviors was obtained by counting all stage 3 seizure-like behaviors displayed.

### Treatment with indomethacin and its toxicological evaluation

Indomethacin (Sigma-Aldrich, St. Louis, MO, USA) was solubilized in 1 % Tris–HCl (pH 8.0) buffer to prepare a stock solution. A primary concentration screen assay was set up for the toxicological evaluation of indomethacin by incubating larvae at six dpf in a 96-well plate (Millipore, USA). Based on our protocol and the protocol described by d’Alençon et al. (2010) [36], indomethacin was tested at seven concentrations (10, 70, 100, 140, 200, 250, and 307 μM). One larva was used *per* well, and 12 larvae were used *per* treatment/concentration groups. After 24 h of incubation in indomethacin, we applied a touch stimulus on the plate to evaluate the startle/escape response of each larva to identify any signs of locomotor impairment and/or toxicity. None of the tested concentrations impaired the startle/escape response of the larvae or caused body deformation, exophthalmos, or death [9, 10, 37]. This assay was performed in duplicates. Then we used 10 and 100 μM indomethacin according to a study by d’Alençon et al. (2010) [36] and 307 μM indomethacin (the maximum dose tested in our assay) before PTZ-induced seizures, and tested the effect of these concentrations on *cox2b* mRNA expression using qPCR. After 24 h of indomethacin exposure, we applied a touch stimulus on the plate to evaluate the startle/escape response of each larva to identify any signs of locomotor impairment and toxicity. The incubation period used for the indomethacin treatment was determined based on a previous AED screening reported by Afrikanova et al. [37]. Zebrafish larvae at six dpf were incubated in 10, 100, and 307 μM indomethacin in petri dishes for 24 h, and then at seven dpf, they were exposed to 15 mM PTZ for 20 min as described above. It is important to highlight that each indomethacin concentration group presented in this study was composed by a different set of animals.

### RNA isolation and reverse transcriptase-PCR

Animals were crioanesthetized and their heads were immediately isolated, quickly frozen in liquid nitrogen, and stored at −80 °C 0.05, 1, 6, 12, 24, and 48 h after PTZ treatment. We pooled 20 larval heads to obtain sufficient material for RNA extraction. A total of five samples (n = 5 for each time point) were used for each group (CG, SG, and indomethacin treatment). Total RNA was extracted using TRIzol^®^ (Invitrogen, Carlsbad, CA, USA) according to the manufacturer’s instructions, and its concentration and quality were determined with the EpochTM spectrophotometer (BioTek, Winooski, VT, USA) and electrophoresis using agarose gels. One microgram of total RNA was reverse transcribed into cDNA using the High Capacity first-strand synthesis system for RT-PCR (Invitrogen, Carlsbad, CA, USA) according to the manufacturer’s instructions. Reactions without RNA were run as negative controls.

### Real-time quantitative PCR

Quantitative PCR was performed using the ABI 7500 Real Time PCR system (Applied Biosystems, Foster City, CA, USA), TaqMan^®^ Universal Master Mix, and TaqMan^®^ Gene Expression Assay (Invitrogen, Carlsbad, CA, USA) for zebrafish (Table 1). All relative quantifications were performed in triplicates and were normalized to the housekeeping gene eukaryotic translation elongation factor 1 alpha 1, like 1 (*eef1a1l1*) [38, 39]. The mRNA level of each target gene (*il1b*, *cox2a*, *cox2b,* and *c*-*fos*) was normalized to the expression level of the housekeeping gene *eef1a1l1* (Table 1). Efficiency of each quantitative real time PCR assay was assessed using standard curves, which were created by measuring five serially diluted cDNA samples in triplicates. Efficiency was calculated according to the following formula: E = 10[−1/slope]. Relative gene expression quantification (RQ) of the SG samples compared with the CG samples (after normalization to the housekeeping gene) was calculated according to the equation RQ = 2 − ∆∆CT described by Livak and Schmittgen [40]. Each reaction was run without cDNA as negative control. Data were analyzed using the SDS 7500 software (Applied Biosystems) to estimate qPCR efficiency and quantify the relative gene expression.

**Table 1:** Information about genes and qPCR assays

Official full name	Gene official symbol	Also known as	Accession	Assay ID
Eukaryotic translation elongation factor 1 alpha 1a	*eef1a1a*	*eef1a1a*	NM_131263.1	Dr03432748_ml
v-fos FBJ murine osteosarcoma viral oncogene homolog Ab	*fosab*	*fos, c-fos*	NM_205569.1	Dr03100809_gl
Interleukin 1-beta	*il1b*	*il1-b*	NM_212844.2	Dr03114369_ml
Prostaglandin-endoperoxide synthase 2a	*ptgs2a*	*cox2a*	NM_153657.1	Dr03080323_ml
Prostaglandin-endoperoxide synthase 2b	*ptgs2b*	*cox2b*	NM_001025504.2	Dr03116323_ml

### Statistical analysis

Data are presented as mean values ± standard error of mean (SEM). Statistical analysis was performed using the GraphPad Prism version 5.0 (GraphPad Software, CA, USA). In all analyses, significance level was set at p ≤ 0.05. Statistical comparisons between two groups were performed using the Mann–Whitney test. Statistical comparisons between three or more groups were performed using one-way analysis of variance (ANOVA) with Bonferroni’s post hoc test.

## Abbreviations

CG: control group
cox2a: cyclooxygenase-2a
cox2b: cyclooxygenase-2b
dpf: days post-fertilization
fos: FBJ murine osteosarcoma viral oncogene homolog
il1b: interleukin-1 beta
NSAID: nonsteroidal anti-inflammatory drugs
ptgs2: prostaglandin-endoperoxide synthase 2
PTZ: pentylenetetrazol
qPCR: quantitative PCR
RT-PCR: reverse transcription PCR
SG: seizure group

## References

[1] Dooley K, Zon LI (2000). Zebrafish: a model system for the study of human disease. Curr Opin Genet Dev.

[2] Shin JT, Fishman MC (2002). From Zebrafish to human: modular medical models. Annu Rev Genomics Hum Genet.

[3] Baraban SC, Taylor MR, Castro PA, Baier H (2005). Pentylenetetrazole induced changes in zebrafish behavior, neural activity and c-fos expression. Neuroscience.

[4] Pineda R, Beattie CE, Hall CW (2011). Recording the adult zebrafish cerebral field potential during pentylenetetrazole seizures. J Neurosci Methods.

[5] Howe K, Clark MD, Torroja CF, Torrance J, Berthelot C, Muffato M, Collins JE, Humphray S, McLaren K, Matthews L (2013). The zebrafish reference genome sequence and its relationship to the human genome. Nature.

[6] Alfaro JM, Ripoll-Gómez J, Burgos JS (2011). Kainate administered to adult zebrafish causes seizures similar to those in rodent models. Eur J Neurosci.

[7] Pichler FB, Laurenson S, Williams LC, Dodd A, Copp BR, Love DR (2003). Chemical discovery and global gene expression analysis in zebrafish. Nat Biotechnol.

[8] Kaufman CK, White RM, Zon L (2009). Chemical genetic screening in the zebrafish embryo. Nat Protoc.

[9] Berghmans S, Hunt J, Roach A, Goldsmith P (2007). Zebrafish offer the potential for a primary screen to identify a wide variety of potential anticonvulsants. Epilepsy Res.

[10] Baxendale S, Holdsworth CJ, Meza Santoscoy PL, Harrison MR, Fox J, Parkin CA, Ingham PW, Cunliffe VT (2012). Identification of compounds with anti-convulsant properties in a zebrafish model of epileptic seizures. Dis Model Mech.

[11] Baraban SC, Löscher W (2014). What new modeling approaches will help us identify promising drug treatments?. Adv Exp Med Biol.

[12] Kwon YS, Pineda E, Auvin S, Shin D, Mazarati A, Sankar R (2013). Neuroprotective and antiepileptogenic effects of combination of anti-inflammatory drugs in the immature brain. J Neuroinflammation.

[13] Gupta P, Khobragade SB, Shingatgeri VM (2014). Effect of various antiepileptic drugs in zebrafish PTZ-seizure model. Indian J Pharm Sci.

[14] Vezzani A, Aronica E, Mazarati A, Pittman QJ (2013). Epilepsy and brain inflammation. Exp Neurol.

[15] Vezzani A, French J, Bartfai T, Baram TZ (2011). The role of inflammation in epilepsy. Nat Rev Neurol.

[16] Xu D, Miller SD, Koh S (2013). Immune mechanisms in epileptogenesis. Front Cell Neurosci.

[17] Aronica E, Crino PB (2011). Inflammation in epilepsy: clinical observations. Epilepsia.

[18] Friedman A, Dingledine R (2011). Molecular cascades that mediate the influence of inflammation on epilepsy. Epilepsia.

[19] Dinarello CA (2002). The IL-1 family and inflammatory diseases. Clin Exp Rheumatol.

[20] Rojas A, Jiang J, Ganesh T, Yang MS, Lelutiu N, Gueorguieva P, Dingledine R (2014). Cyclooxygenase-2 in epilepsy. Epilepsia.

[21] Samad TA, Moore KA, Sapirstein A, Billet S, Allchorne A, Poole S, Bonventre JV, Woolf CJ (2001). Interleukin-1beta-mediated induction of Cox-2 in the CNS contributes to inflammatory pain hypersensitivity. Nature.

[22] Claycomb RJ, Hewett SJ, Hewett JA (2012). Neuromodulatory role of endogenous interleukin-1β in acute seizures: possible contribution of cyclooxygenase-2. Neurobiol Dis.

[23] Molina-Holgado E, Ortiz S, Molina-Holgado F, Guaza C (2000). Induction of COX-2 and PGE(2) biosynthesis by IL-1beta is mediated by PKC and mitogen-activated protein kinases in murine astrocytes. Br J Pharmacol.

[24] Neeb L, Hellen P, Boehnke C, Hoffmann J, Schuh-Hofer S, Dirnagl U, Reuter U (2011). IL-1β stimulates COX-2 dependent PGE_2_ synthesis and CGRP release in rat trigeminal ganglia cells. PLoS One.

[25] Novoa B, Figueras A (2012). Zebrafish: model for the study of inflammation and the innate immune response to infectious diseases. Adv Exp Med Biol.

[26] Langevin C, Aleksejeva E, Passoni G, Palha N, Levraud JP, Boudinot P (2013). The antiviral innate immune response in fish: evolution and conservation of the IFN system. J Mol Biol.

[27] Trede NS, Langenau DM, Traver D, Look AT, Zon LI (2004). The use of zebrafish to understand immunity. Immunity.

[28] Meeker ND, Trede NS (2008). Immunology and zebrafish: spawning new models of human disease. Dev Comp Immunol.

[29] Minami M, Kuraishi Y, Yamaguchi T, Nakai S, Hirai Y, Satoh M (1990). Convulsants induce interleukin-1 beta messenger RNA in rat brain. Biochem Biophys Res Commun.

[30] Järvelä JT, Lopez-Picon FR, Plysjuk A, Ruohonen S, Holopainen IE (2011). Temporal profiles of age-dependent changes in cytokine mRNA expression and glial cell activation after status epilepticus in postnatal rat hippocampus. J Neuroinflammation.

[31] Ishikawa TO, Griffin KJ, Banerjee U, Herschman HR (2007). The zebrafish genome contains two inducible, functional cyclooxygenase-2 genes. Biochem Biophys Res Commun.

[32] Grosser T, Yusuff S, Cheskis E, Pack MA, FitzGerald GA (2002). Developmental expression of functional cyclooxygenases in zebrafish. Proc Natl Acad Sci USA.

[33] Vieira MJ, Perosa SR, Argaãaraz GA, Silva JA, Cavalheiro EA, Graça Naffah-Mazzacoratti M (2014). Indomethacin can downregulate the levels of inflammatory mediators in the hippocampus of rats submitted to pilocarpine-induced status epilepticus. Clinics (Sao Paulo).

[34] Westerfield M (2000). The zebrafish book. A guide for the laboratory use of zebrafish (Danio rerio).

[35] Kimmel CB, Ballard WW, Kimmel SR, Ullmann B, Schilling TF (1995). Stages of embryonic development of the zebrafish. Dev Dyn.

[36] d’Alençon CA, Peña OA, Wittmann C, Gallardo VE, Jones RA, Loosli F, Liebel U, Grabher C, Allende ML (2010). A high-throughput chemically induced inflammation assay in zebrafish. BMC Biol.

[37] Afrikanova T, Serruys AS, Buenafe OE, Clinckers R, Smolders I, de Witte PA, Crawford AD, Esguerra CV (2013). Validation of the zebrafish pentylenetetrazol seizure model: locomotor versus electrographic responses to antiepileptic drugs. PLoS One.

[38] McCurley AT, Callard GV (2008). Characterization of housekeeping genes in zebrafish: male-female differences and effects of tissue type, developmental stage and chemical treatment. BMC Mol Biol.

[39] Tang R, Dodd A, Lai D, McNabb WC, Love DR (2007). Validation of zebrafish (*Danio rerio*) reference genes for quantitative real-time RT-PCR normalization. Acta Biochim Biophys Sin (Shanghai).

[40] Livak KJ, Schmittgen TD (2001). Analysis of relative gene expression data using real-time quantitative PCR and the 2(-Delta Delta C(T)) Method. Methods.

